# *Ex situ* and *in situ* data for endangered livestock breeds in Spain

**DOI:** 10.1016/j.dib.2021.106805

**Published:** 2021-02-04

**Authors:** Rafael De Oliveira Silva, Oscar Cortes Gardyn, Sipke-Joost Hiemstra, Joao G. Oliveira Marques, Michèle Tixier-Boichard, Dominic Moran

**Affiliations:** a Global Academy of Agriculture and Food Security, The University of Edinburgh, Easter Bush Campus, Midlothian, EH25 9RG Edinburgh, UK; b Animal Science Department. Faculty of Veterinary, University Complutense of Madrid, 28040 Madrid, Spain; c Centre for Genetic Resources, Wageningen University & Research, Wageningen Campus, Droevendaalsesteeg 1, 6700 AH Wageningen, The Netherlands; d University Paris-Saclay, INRAE, AgroParisTech, GABI, Jouy-en-Josas 78350, France

**Keywords:** Animal genetic resources, Gene banks, Cryoconservation, *In situ* conservation

## Abstract

Improvements in *ex situ* storage of genetic and reproductive materials offer an alternative for endangered livestock breed conservation. This paper presents a dataset for current *ex situ* collections and *in situ* population for 179 Spanish livestock breeds of seven species, cattle, sheep, pig, chicken, goat, horse and donkey. *Ex situ* data was obtained via survey administered to 18 functioning gene banks in Spain and relates to the reproductive genetic materials (semen doses) of 210 livestock breeds distributed across the gene banks. *In situ* data combines CENSUS information with linear regression techniques and relates to the geographic distribution of 179 Spanish autochthonous livestock breeds (2009-2018), and *in situ* population projections and extinction probabilities (2019-2060). We use a decision variable defining an “acceptable level of risk” that allows decision makers to specify tolerable levels of *in situ* breed endangerment when taking *ex situ* collection and storage decisions.

## Specifications Table

**Table:** 

Subject	Animal Science
Specific subject area	Animal genetic resources, *ex situ* conservation
Type of data	Table
How data were acquired	The data were obtained via surveys, official CENSUS data and mathematical modelling. Data were converted into .xlsx format and analysised in Matlab.
Data format	Raw Analysed Filtered
Parameters for data collection	Gene banks identification were anonymized.
Description of data collection	Genetic material data, in number of semen doses of 0.25mL, were obtained via surveys with gene banks that provided most recent (2018) information on stored materials. *In situ* data, in terms of the number of breeding males and females, were provided by the National Information System of Spanish Livestock Breeds (ARCA), held by the Ministry of Agriculture, Fisheries and Food and used for linear regression analysis.
Data source location	Institution: Ministry of Agriculture, Fisheries and Food City/Town/Region: Madrid Country: Spain Primary data: Number of breeding males and females, and their geographic distributions were provided by the National Information System of Spanish Livestock Breeds (ARCA https://www.mapa.gob.es/es/ganaderia/temas/zootecnia/razas-ganaderas/
Data accessibility	Repository name: Mendeley data Data identification number: https://dx.doi.org/10.17632/mn26mrb243.2 Direct link: https://data.mendeley.com/datasets/mn26mrb243/3[1].
Related research article	Rationalizing *ex situ* collection of reproductive materials for endangered livestock breed conservation. Ecological Economics. https://dx.doi.org/10.1016/j.ecolecon.2020.106916

## Value of the Data

• The data contains the most up to date information of *ex situ* collections and *in situ* populations of Spanish livestock breeds. The data provides valuable information on the prioritization of endangered breeds to support national biodiversity conservation efforts for livestock.
• The data will be useful for decision makers planning *ex situ* collections in Spain, including universities, livestock industry and government.
• The data can be used for the rationalization, planning and investment decision making for *ex situ* collections and *in situ* conservation efforts for biodiversity conservation.

## 1 Data Description

Table 1 describes the number of semen doses of 210 livestock breeds currently in 18 Spanish gene banks (GB) in 2018. “Species – breed” represents a livestock species, cattle, chicken, donkey, goat, horse, pig and ship, followed by the breed name, e.g., “cattle – avilena”. The values in the GB1 to GB18 rows represent the number of semen doses, in straws of 0.25mL that are stored in the Spanish gene banks.

Table 2 describes the geographic location of Spanish livestock breeds. The rows “a_coruna”, “alava” to “zaragoza” are 52 Spanish provinces and values in the rows represent the number of breeding males available for *ex situ* collections (data for 2018). Table 3 shows the travel distances (in km) between the 52 Spanish provinces considered in the study. Table 4 shows the *in situ* CENSUS data [3] for breeding males (potential semen donors). The columns “2009” to “2018” are the years for which the CENSUS data is available, while the values in the rows are the number of breeding males for each of the livestock breeds in Spain. Table 5 shows the projected number of breeding females from 2009 to 2060 using a linear regression and CENSUS data. The columns “a” and “b” are coefficients of the linear regression y = a +b*x. Table 6 shows the lower bound of the projected number of breeding females (2009 to 2060) from the linear regression with 95% confidence. Table 7 shows the upper bound of the projected number of breeding females (2009 to 2060) from a linear regression with 95% confidence. Table 8 describes the annual probability of critical risk under CTC (critical) scenario, i.e., it shows the probability of the number of breeding females (Nbf) being fewer than 100 animals using Eq. (1). Table 9 shows the annual probability of endangerment risk under EDG (endangered) scenario, i.e., the probability of the number of breeding females (Nbf) being fewer than 1000 animals using Eq. (1). Table 10 describes information on FAO [2] recommended number of donors (α), recommended number of doses per donor of species (β), equivalent number of straws (0.25mL) per semen dose, total doses per collection (µ) (equivalent in straws), for cattle, goat, sheep, equine (horse and donkey), pig and chicken.

## 2 Experimental Design, Materials and Methods

Census data were provided by the National Information System of Spanish Livestock Breeds (ARCA) held by the Ministry of Agriculture, Fisheries and Food [3] containing time series for breeding females and males of all autochthonous livestock breeds from 2009 to 2018. The data and the geographic distribution of the animals is uploaded to the ARCA web portal annually by the breed associations. The data (Table 1-3) cover all Spanish autochthonous breeds for seven livestock species (the number of breeds classified as non-endangered/endangered for each species in parentheses), cattle (8/31), sheep (36/8), goats (20/3), pigs (3/12), chicken (1/16), equine (horse and donkey) (1/14).

A survey (See Supplementary Information) was also distributed to 8 autonomous community gene banks and another 10 gene banks selected by geographical location and/or biological material stored. The survey gathered information on species germplasm and breeds and economic data related to gene bank maintenance costs and ex situ collection costs.

To plan *ex situ* collections from 2018 to 2060 we used data for 210 livestock breeds, of which 179 are Spanish autochthonous breeds distributed across 52 provinces, 18 gene banks based in 15 different locations. We used census data from 2009 to 2018 for the linear regression and the production bounds calculated using Matlab [4]. The coeficients for the linear regression are presented in Table 5.

The lower and upper bounds of the *in situ* projections, respectively L_t,sb_ and U_t,sb_ (Table 6 and 7, respectively), representing the number of breeding females in year t of Spanish breed sb, is used to calculated the extinction probabilities (Riskt,sb) as follows

(1) $$Risk_{t,sb}\left\{ \begin{array}{c} 1,\;\text{if}\;N\;-\;U_{t,sb}>0\\ 0,\;if\;N-L_{t,sb}>0\\ \frac{N-L_{t,sb}}{U_{t,sb}-L_{t,sb}},\;otherwise\\ \; \end{array}\right.$$

Where N is equal 100 for CTC scenario, and 1000 for EDG scenario (Table 8 and 9).

Matlab code for generating and plotting a linear regression for *in situ* populations, i.e., Tables 5-7) is presented as Supplementary Information.

## Ethics Statement

Participation in the survey was voluntary and respondents were informed that the data would be analyzed anonymously. In participating in the survey and submitting the questionnaire each respondent (gene bank manager) gave their informed consent.

## Declaration of Competing Interest

The authors declare that they have no known competing financial interests or personal relationships that have or could be perceived to have influenced the work reported in this article.
